# ID 344 - Proposta de Estruturação de Processo de Tutoria para a Elaboração de Diretrizes Clínico-Assistenciais para o SUS

**DOI:** 10.5327/2237-9622.2025.v34s1.344

**Published:** 2025-11-25

**Authors:** Muriel Primon de Barros, Cintia Laura Pereira Araujo, Roseana Boek Carvalho, Marina Petrasi Guahnon, Ana Paula Blankenheim, Bárbara Cristiane da Silva, Bruna Marmett, Rodrigo Pereira de Almeida, Marta da Cunha Lobo Souto Maior, Gilson Pires Dorneles, Suena Medeiros Parahiba, Maicon Falavigna

**Keywords:** diretrizes clínico-assistenciais, processo de tutoria, núcleos de Avaliação de Tecnologias em Saúde (Nats)

## Abstract

**Introdução:**

As diretrizes clínico-assistenciais do Sistema Único de Saúde (SUS) brasileiro, como os Protocolos Clínicos e Diretrizes Terapêuticas (PCDT), são elaboradas por Núcleos de Avaliação de Tecnologias em Saúde (Nats) e avaliadas pela Comissão Nacional de Incorporação de Tecnologias no Sistema Único de Saúde (Conitec) – os quais orientam o diagnóstico, o tratamento e o acompanhamento de pacientes com diversas condições de saúde – e devem ser seguidas pelos profissionais de saúde e gestores no SUS. Ações de tutorias são importantes para o aperfeiçoamento metodológico de profissionais responsáveis pelo desenvolvimento de diretrizes, visando à capacitação dos Nats com uma melhor gestão e à melhoria contínua no desenvolvimento desses documentos. O objetivo deste estudo foi apresentar os resultados do mapeamento e aprimoramento do processo de tutoria de profissionais para elaboração de diretrizes clínico-assistenciais para o SUS.

**Método:**

A inter-relação dos processos envolvidos foi identificada e os conjuntos de ações foram agregados em macroprocessos, processos, subprocessos, atividades e tarefas, considerando as recomendações das diretrizes metodológicas de elaboração de diretrizes clínicas publicadas pelo Ministério da Saúde e dos padrões recomendados pelo (GIN) e pelo (IOM). O processo de tutoria foi mapeado utilizando a ferramenta (BPMN), e os fluxogramas foram construídos com a ferramenta .

**Resultados:**

Aspectos importantes do processo no desenvolvimento de diretrizes clínicas foram mapeados, como a interrelação dos processos, que identificou as partes envolvidas e suas respectivas responsabilidades, relacionando o demandante com processos gerenciais, o Nats executor com os processos finalísticos e tutores com os processos de suporte. Os conjuntos de ações foram identificados, agregados e distribuídos entre as partes envolvidas, resultando na elaboração de um fluxograma de todo o processo de tutoria e sete fluxogramas dos subprocessos relacionados: 1) delimitação do escopo das diretrizes; 2) definição das perguntas PICO; 3) definição de estratégia de busca e seleção de estudos; 4) extração de dados e síntese da evidência; 5) avaliação da certeza do corpo da evidência; 6) elaboração e graduação das recomendações pelo sistema GRADE (do inglês, ); e, 7) redação do texto das diretrizes.

**Conclusão:**

Os fluxogramas elaborados destacam a metodologia estruturada e o apoio contínuo fornecido por programas de tutores aos Nats para expandir o número de núcleos capacitados para o desenvolvimento de diretrizes. Pelo mapeamento realizado, será possível gerenciar e analisar continuamente o desempenho do processo, modificar atividades que não agregam valor, evitar falhas e manter o foco nas necessidades dos Nats no desenvolvimento de diretrizes. Esse suporte é crucial para garantir que cada diretriz clínica seja desenvolvida com rigor científico e de acordo com as políticas do SUS.

